# How to address the challenges of evaluating treatment benefits-risks in rare diseases? A convergent mixed methods approach applied within a Merkel cell carcinoma phase 2 clinical trial

**DOI:** 10.1186/s13023-018-0835-1

**Published:** 2018-06-18

**Authors:** Murtuza Bharmal, Isabelle Guillemin, Alexia Marrel, Benoit Arnould, Jérémy Lambert, Meliessa Hennessy, Fatoumata Fofana

**Affiliations:** 1 Merck KGaA, Frankfurter Str. 250, Postcode F135/301, 64293 Darmstadt, Germany; 2 Mapi, Patient-Centered Outcomes, Lyon, France; 3 EMD Serono, Immuno-Oncology, Billerica, MA USA; 4 Mapi, Patient-Centered Outcomes, Leiden, the Netherlands

**Keywords:** Mixed methods research, Merkel cell carcinoma, Qualitative outcomes, Quantitative outcomes, Treatment meaningfulness

## Abstract

**Background:**

Demonstrating treatment benefits within clinical trials in the context of rare diseases is often methodologically and practically challenging. Mixed methods research offers an approach to overcome these challenges by combining quantitative and qualitative data, thus providing a better understanding of the research question. A convergent mixed methods design in the context of Merkel cell carcinoma, a rare skin cancer, was used during the JAVELIN Merkel 200 trial (NCT02155647).

**Methods:**

Nine patients receiving avelumab in the JAVELIN Merkel 200 trial were interviewed at baseline prior to receiving study treatment, and at 13 weeks and 25 weeks after first avelumab administration. Key concepts of interest identified from the baseline interviews were physical functioning, fatigue/energy, and pain. Patient perceptions of the overall change in their cancer-related health status since starting study treatment were also recorded. During qualitative analysis, at each time-point, each concept of interest was assigned a category describing the trend in change (e.g. newly emerged, no change/stable, improved, worsened, ceased/disappeared). In parallel, patients’ tumour status was determined by the clinical overall response status as per the clinical trial protocol.

**Results:**

A high concordance between patient-reported qualitative data and assessed tumour response was observed. All eight patients who clinically improved had perceived a subjective improvement in their disease since the beginning of the study; the single patient whose disease worsened had a perceived deterioration. Patient perceived benefit in physical functioning, fatigue/energy and pain was subsequent to the measured change in clinical status as assessed by tumour response. This suggests that patient-reported assessment should be examined over the long term in order to optimally capture meaningful treatment effect.

**Conclusion:**

Embedding qualitative research in clinical trials to complement the quantitative data is an innovative approach to characterise meaningful treatment effect. This application of mixed methods research has the potential to overcome the hurdles associated with clinical outcomes assessment in rare diseases.

## Background

The evaluation of the benefits-risk assessment of orphan drugs brings numerous methodological and practical issues [1]. Amongst these, evaluating patient perspective is particularly arduous and challenging in rare diseases. In addition to facing small sample sizes, patient-reported outcome (PRO) questionnaires specific for rare diseases often do not exist, and generic questionnaires lack specificity and responsiveness to demonstrate treatment effects. The standardized procedures for PRO questionnaire development to support labelling claims, and the qualification for drug development tools recently released by the FDA [2] are hardly feasible in the context of rare diseases; and similarly, the FDA roadmap is difficult to apply to orphan drugs [3]. First, because the number of participants enrolled in clinical trials is small, trials are often underpowered to enable statistically significant and/or robust conclusions from PRO questionnaire results to be drawn. Second, defining a specific endpoint common to the population that will allow the assessment of a treatment benefit is known to be complex in most if not all diseases. This is even more complex in the context of a rare disease, due to the heterogeneity of the patients’ profiles, in particular with regard to age and disease stage. Third and last, the best candidates for concepts of interest used to show a meaningful treatment effect are often not known at the time of start of the clinical trial due to the lack of knowledge of natural history of these diseases and the novelty of the drugs being assessed. This is especially true for rare diseases where randomized controlled clinical trials are usually not possible, thus preventing comparison of PRO data within and across trials. Consequently, conventional PRO methods used to demonstrate a meaningful treatment benefits-risks assessment and support labelling claims may be challenged in the context of rare diseases.

Despite recent emphasis on the importance of evaluating disease-related symptoms, treatment-related symptoms and physical functioning directly from oncology patient trials, patient-reported outcomes that may support drug labelling are still rarely implemented, particularly in the US [4, 5].

While mixed methods research is recognised and well established in the social and behavioural sciences [6], it has only recently emerged in clinical research [7]. Mixed method research provides the advantages of qualitative research, which includes a large and rich amount of explorative data allowing expression of patients’ voices and exploration of the disease and its management. Mixed methods research offers a methodological tool to overcome the challenges of PRO assessment in rare diseases, while at the same time preserving the advantages of reliability, evidence generation and hypothesis testing typical of quantitative research [8].

Merkel cell carcinoma (MCC) is a rare, aggressive cutaneous malignancy [9]. MCC has a high recurrence rate and rapidly metastasizes, often leading to limited 5-year survival [9–12]. In ≈80% of cases, MCC is associated with Merkel cell polyomavirus infection [13]. Risk factors associated with increased risk of developing MCC include excessive sun exposure, a compromised immune system, light skin colour, older age, and history of skin cancer [9, 14]. The incidence rate of MCC varies across countries, with 0.13 per 100,000 between 1995 and 2002 in Europe, and 0.79 cases per 100,000 in the United States in a 2011 report from the Surveillance, Epidemiology and End Results (SEER) program [15, 16]. Avelumab is a human anti–PD-L1 IgG1 monoclonal antibody that inhibits interaction between PD-L1 and PD-1 [17]. Avelumab has shown efficacy and an acceptable safety profile in a phase 2 clinical trial (JAVELIN Merkel 200; NCT02155647) in metastatic MCC [18], and has recently been approved by both the FDA and the EMA for the treatment of patients 12 years and older with metastatic MCC.

Limited information is currently available on the everyday lives of patients with MCC, and there are no published qualitative data on how patients feel, function and survive on an everyday basis [19].

During conduct of the JAVELIN Merkel 200 clinical trial there were no specific questionnaires to assess the quality of life of patients with MCC within clinical trials. The Functional Assessment of Cancer Therapy – General (FACT-G) and European Organisation for Research and Treatment of Cancer -Quality of Life (EORTC QLQ-C30) questionnaires are the most extensively used instruments in oncology [20, 21]. FACT-M is a melanoma-specific instrument derived from FACT-G [20, 22]. Psychometric evidence of good reliability and validity are available in different settings for the FACT-G and the EORTC-QLQ-C30 [23–25] and in melanoma for the FACT-M [20, 26]. The FACT-M questionnaire was used in the phase 2 JAVELIN Merkel 200 clinical trial to quantitatively assess the impact of MCC on patients, and appropriateness for FACT-M use in MCC has been reported [27].

To overcome the challenge of evaluating meaningfulness of treatment effect from a patient-reported perspective in the context of this rare cancer, a mixed methods approach that followed a convergent design was used [28]. In this study, qualitative interviews were performed with the patients participating in the JAVELIN Merkel 200 trial; in parallel, patients’ overall response by Independent Endpoint Review Committee (IERC) per Response Evaluation Criteria In Solid Tumors version 1.1 (RECIST) was determined clinically to report patients’ tumour response status [29]. Data from both the patient interviews and the clinical evaluations were then merged to look for correspondence between the qualitative outcomes data and the clinical and patient-reported quantitative outcomes data.

## Methods

### Study design

The single-arm, open-label, multicentre, international phase 2 JAVELIN Merkel 200 trial (NCT02155647) was conducted to evaluate the efficacy and safety of avelumab in patients with distant metastatic MCC. Patients enrolled in the first part of the trial were adults aged at least 18 years who had chemotherapy-refractory, with histologically confirmed MCC and had failed at least one line of chemotherapy; the second part of the trial is on-going at the time this manuscript is prepared with adults aged at least 18 years who are treatment naïve in the context of metastatic MCC. The present manuscript reports results from the first part of the trial. Patients received avelumab at a dose of 10 mg/kg as a 1-h intravenous infusion every two weeks until significant clinical deterioration, unacceptable toxicity, or any protocol-specified criterion for withdrawal from the trial or trial drug was fulfilled. The primary endpoint was confirmed objective response (complete response or partial response) assessed according to RECIST version 1.1 by an independent review committee. Details on the definitions of these inclusion criteria as well as study design, including efficacy and safety endpoints are reported elsewhere [18]. Assessment of patient-perceived experience of the disease and treatment benefit was ranked as an exploratory endpoint and assessed through the use of patient-reported outcome questionnaires and patient interviews as described below.

Upon recruitment, all patients were invited to participate in optional qualitative interviews. Patients agreeing to participate were interviewed during the screening period, before first administration of the study treatment.

The clinical trial protocol, including description of the qualitative interviews, was approved by all relevant independent ethics committees and institutional review boards and was conducted in accordance with the Declaration of Helsinki and Good Clinical Practice. Patients provided written informed consent before any trial-related activity. Patients who agreed to be interviewed indicated their willingness to participate within the informed consent form.

Data reported in this study are based on the protocol specified analysis with a cut-off date on 3 March 2016, six months after start of study treatment of the last patient.

### Quantitative variable: Clinical status assessment

Overall response by IERC per RECIST version 1.1 was used to assess patients’ clinical tumour status [30]. Improved clinical status corresponded to partial or complete response (P/CR), unchanged clinical status corresponded to stable disease (SD), and worsened clinical status to progressive disease (PD).

### Quantitative variable: FACT-M assessment

FACT-M data were collected electronically at sites throughout the treatment period (at baseline, week seven and then every six weeks) and at the end-of-treatment visit.

The FACT-M comprises 51 items grouped into nine multi-item scores, including six subscales scores and three summary scores [20, 26]. The six subscales consist of four subscales from the FACT-G (Physical well-being [PWB], Social well-being [SWB], Emotional well-being [EWB], Functional well-being [FWB]), one Melanoma scale, and one Melanoma surgery scale. The three summary scores include the FACT-M Trial Outcome Index (TOI), the FACT-G total score, and the FACT-M total score.

### Qualitative variable: Patient interviews

Qualitative patient interviews were conducted to collect comprehensive qualitative information on the impact of MCC and its treatments (e.g. radiotherapy or chemotherapy) on patients’ everyday lives, as well as patients’ experience with avelumab during the trial.

Qualitative interviews were optional; patients were invited to participate as they consented to the trial, but were free to accept or refuse to participate in the qualitative interviews. Upon acceptance, patients were offered to be interviewed at three pre-defined time-points during the clinical trial: at baseline prior to receiving the study treatment avelumab and at study Week 13 and Week 25 (i.e. 12 weeks and 24 weeks after first administration of avelumab during study Week 1) if they had not discontinued the study prior to that time points.

The aim of baseline interviews was to gain a comprehensive picture of patients’ lives with MCC, covering the period before diagnosis, at the time of diagnosis of MCC, following diagnosis, and through to commencement of treatment [30]. Follow-up interviews documented the change (improvement, stability, or worsening) in disease status following treatment initiation, as well as the patient’s experience of treatment. Upon recruitment in the trial, all patients were invited to participate in these optional qualitative interviews. Patients could be provided with the results of scan exams or blood tests by the clinical team during their scheduled assessments visits every 6 weeks. Written informed consent was obtained from all the patients who agreed to be interviewed.

Trained interviewers, external to the clinical team and native-speakers of the patient’s local language, performed the interviews. Phone interviews lasted approximately 30 min and were audio-recorded and transcribed verbatim. Interviews were conducted using an interview guide specifically developed for this study and were unique for each of the time-points. For the baseline interviews, non-directive interview techniques and open-ended questions were used to let the interviewees answer spontaneously. If necessary, specific queries were used to collect in-depth knowledge and information from the patients. For the follow-up interviews, an overall open-ended question was used to inquire about a patients’ assessment of their health status after receiving the study treatment and to follow up on what had changed in terms of signs and symptoms and related-impact since the patient started the study and received avelumab.

Interview transcripts were analysed with the Atlas.ti qualitative software package [31], using a thematic analysis approach [32, 33]. From all identified concepts and sub-concepts in the baseline interviews, the following concepts were selected based on their clinical relevance [5, 34]: physical functioning, fatigue/energy, and pain. The progression of these concepts throughout the study was specifically explored at Week 13 and at Week 25 during the analysis. Each interview was qualitatively analysed at the individual level. At each time-point, each concept of interest was assigned a category describing the trend in change that may have occurred between baseline and Week 13, between Week 13 and Week 25, and since starting study treatment. Categories were adapted from Saldana [35] and included newly emerged, no change/stable, improved, worsened, ceased/disappeared, missing, and turning point (i.e. experience or event that may significantly alter the perceptions and/or life course of the patient since baseline). In addition, the concept of overall change in cancer status since starting study treatment was assessed by asking each of the patients the following question “Has your cancer changed since you started the study and received the study treatment?” at the beginning of the interview. Patients’ status (i.e. improved, worsened, stable or new) and quotes corresponding to each of the concepts were extracted and used to identify how patients described their health status in their own words.

### Analysis

#### Patient population

The description of baseline characteristics were conducted to characterize the interviewed patient population.

#### Longitudinal FACT-M data

For the purpose of mixed-methods analysis, a sample of FACT-M items/scores was selected based on the similarity with the selected qualitative concepts (Table 1). For each item/score, the change from baseline to Week 25 was calculated and interpreted as follows: a positive change was associated with an improvement, a null change was associated with no change and a negative change was associated with a worsening.

**Table 1:** Sample of FACT-M items/scores corresponding to the selected qualitative concepts

Qualitative concept	Corresponding FACT-M item/score
Physical functioning	Physical Well-being score
	Functional Well-being score
Pain	GP4. I have pain
	M1. I have pain at my melanoma site or surgical site
	M5. I have aches and pain in my bones
	M13. Movement of my swollen area is painful
Fatigue	HI7. I feel fatigued

#### Longitudinal qualitative data

For longitudinal qualitative interviews, coding was first performed at the individual level to explore the experience of each patient over time. For each key concept identified at the baseline analysis and probed at the follow-up interviews, a category was assigned to show changes in concepts that occurred between the two time points (Newly emerged, Not changed/Stable, Improved, Worsened, Ceased/Disappeared, Missing, Turning point) [36]. Each of the concepts probed during the follow-up interviews (Week 13 and Week 25) was categorized and compared to the baseline coding [36].

An analysis was then conducted on the pooled population at each of the follow-up time points to document the experience of the study population over time.

## Results

### Patient population

Of the 88 patients with metastatic MCC whose disease progressed after latest chemotherapy and who were enrolled in the JAVELIN Merkel 200 clinical trial Part A, 19 accepted to be interviewed at baseline prior to receiving study treatment; of those 19 patients, 12 were interviewed at Week 13, and 10 at Week 25. A total of nine patients were interviewed at all three time-points and had a clinical status assessment. This constituted the sample for the mixed methods approach (qualitative and quantitative analysis). The majority were male (*n* = 7; 78%) from the United States (*n* = 8; 88%), with a mean age of 70.8 ± 9.8 years (Table 2). Most patients (n = 8; 88%) were categorized as having an improved tumour response, with eight PRs/CRs at both Week 13 and Week 25 time-points. One patient (11%) had a worsened tumour response with a PD.

**Table 2:** Patient characteristics and clinical outcomes

Variable	Population (*N* = 9)
Age (years)	
Mean (Sd.)	70.8 (9.8)
Median	71.0
Min - Max	55.0–85.0
Gender (n)	
Male	7
Female	2
Country (n)	
Germany	1
USA	8
Overall response by IERC per RECIST at Week 13 / Week 25 (n)	
Partial / Complete responder	8 / 8
Stable responder	0 / 0
Progressive disease	1 / 1

*Sd* standard deviation, *USA* United States of America, *IERC* Independent Endpoint Review Committee, *RECIST* Response Evaluation Criteria In Solid Tumors version 1.1

### Mixed methods research analysis: Qualitative findings in relation with quantitative findings

The progression of each of the selected concepts of interest was correlated with the patients’ clinical status at Week 13 and at Week 25. In addition, the change in FACT-M items showed that these patients’ HRQoL showed no deterioration overall. The results are reported in the sub-sections below. The overall progression trend of the concepts since starting the study treatment is reported in Tables 3, 4 and 5, and the corresponding patient narratives are summarized in Table 6.

**Table 3:** Progression trend of the ‘Overall change in patients’ perception of their cancer’ concept since starting study treatment up to Week 25 and its correspondence with overall response by IERC per RECIST

Patient #	Overall response by IERC per RECIST^a^	Overall change in patients’ perception of their cancer
1	Partial/complete responder	Improvement
2	Partial/complete responder	Improvement
3	Partial/complete responder	Improvement
4	Partial/complete responder	Improvement
5	Partial/complete responder	Improvement
6	Partial/complete responder	Improvement
7	Partial/complete responder	Improvement
8	Partial/complete responder	Improvement
9	Progressive disease	No change (still worsened)

*IERC* Independent Endpoint Review Committee, *RECIST* Response Evaluation Criteria In Solid Tumors version 1.1

^a^Overall response by IERC per RECIST identical at both Week 13 and Week 25 for all patients

**Table 4:** Progression trend of the ‘Physical functioning’ concept since starting study treatment up to Week 25 and its correspondence with quantitative assessments

Patient #	Qualitative assessment	Quantitative assessment		
	Physical functioning	Overall response by IERC per RECIST^a^	Change in FACT-M score related to Physical functioning	
			Physical Well-being	Functional Well-being
1	Improvement	Partial/complete responder	No change	Improvement
2	Improvement	Partial/complete responder	Worsening	Worsening
3	Improvement	Partial/complete responder	No change	Worsening
4	Improvement	Partial/complete responder	Improvement	Improvement
5	No change (no impact)	Partial/complete responder	No change	Improvement
6	No change (no impact)	Partial/complete responder	No change	Improvement
7	No change (no impact)	Partial/complete responder	Improvement	Improvement
8	Improvement	Partial/complete responder	NC	NC
9	No change (no impact)	Progressive disease	Worsening	Improvement

*NC* Not computed, due to missing baseline data, *IERC* Independent Endpoint Review Committee; RECIST: Response Evaluation Criteria In Solid Tumors version 1.1

^a^Overall response by IERC per RECIST identical at both Week 13 and Week 25 for all patients

**Table 5:** Progression trend of the ‘Fatigue’ and ‘Pain’ concepts since starting study treatment up to Week 25 and its correspondence with quantitative assessments

Patient #	Qualitative assessment		Quantitative assessment					
			Overall response by IERC per RECIST^a^	Change in FACT-M item related to Fatigue	Change in FACT-M item related to Pain			
	Fatigue	Pain		HI7. I feel fatigued	GP4. I have pain	M1. I have pain at my melanoma site or surgical site	M5. I have aches and pains in my bones	M13. Movement of my swollen area is painful
1	No change (still tired)	No change (no pain)	Partial/complete responder	No change	Improvement	No change	Worsening	No change
2	Improvement	No change (no pain)	Partial/complete responder	No change	Worsening	Improvement	No change	No change
3	No change (still tired)	No change (no pain)	Partial/complete responder	No change	Worsening	No change	Worsening	No change
4	Improvement	ND	Partial/complete responder	Improvement	No change	No change	No change	No change
5	ND	No change (no pain)	Partial/complete responder	No change	No change	Improvement	Improvement	No change
6	Improvement	Improvement	Partial/complete responder	No change	No change	No change	Worsening	No change
7	Improvement	Improvement	Partial/complete responder	Improvement	Improvement	Improvement	Worsening	No change
8	ND	Improvement	Partial/complete responder	NC	NC	NC	NC	NC
9	ND	No change (still pain)	Progressive disease	Worsening	Worsening	No change	No change	No change

*NC* Not computed, due to missing baseline data, *ND* Not determined, trend not possible to determine as data was not reported spontaneously by the patient and was not probed at one of the time-point interviews, *IERC* Independent Endpoint Review Committee; RECIST: Response Evaluation Criteria In Solid Tumors version 1.1

^a^Overall response by IERC per RECIST identical at both Week 13 and Week 25 for all patients

**Table 6:** Excerpts of patient quotes illustrating the status of the selected concepts

Clinical status^a^	Progression of concepts at Week 13 or Week 25 interviews^b^	Patients’ quotes extracted from Week 13 or Week 25 interviews			
		Perceived change in cancer^c^	Physical functioning	Fatigue/Energy	Pain
Responders (n = 8 patients)	Improved	“It’s like much, much better. I’m doing very well. I’m doing especially well. Well, it happens to be that one of my tumours is something that I can touch, and I can tell it’s getting smaller, and the CAT scans I’m getting every six weeks are indicating the same thing.” (Week 25)	“I’m doing things that I haven’t done in a long time just like being able to go out and walk” (Week 25)	“I have more energy... I mean, I had energy when I started, but it was more of a forced energy, now it’s not a... It’s like I forced myself to do things, so that I would keep going. Now I don’t need to force myself […] Sometimes I have some fatigue, but it’s not bad, I mean, it’s just a mild fatigue” (Week 25)	“well, I have, I’m still having some pain in my back, but it has lessened since I started the infusions” (Week 13)
	Unchanged	“not to my knowledge. I’m due for another set of scans next Monday, so…”	“I feel fine and continue to do what I can. I mean, I am 74 so, you know, I don’t do, unlike former President Bush, I don’t parachute jumping and stuff like that on my birthday, but, you know, I do not feel limited due to the cancer and what I do in terms of be daily activities” (Week 25)	“I’m not that strong, I mean I’m up and about all day and I work for a couple of hours then I come in and lay down and rest for about 30 min and then go out and work some more […] I’m draggy, I wear out quick” (Week 13)	“No, no pain whatsoever” (Week 25)
	Worsened	No patients	“I wanted to dance with my wife, and I could get maybe one dance in and then I might have to rest for 45 min before I can dance again, […] I’m down, I can’t probably walk much less, getup and dance” (Week 13)		No patients
Progressive disease (n = 1 patient)	Improved	No patients	No patients	N/A	No patients
	Unchanged	No patients	“Physically I’m still fairly strong in what I’m doing, and active as much as I can be […] at this point in time noticed any drop off in my physical capabilities.” (Week 13)	N/A	No patients
	Worsened	“To the best of my knowledge it has metastasized just slightly, based on the last scan I had. Still located in the abdominal area and esophagus area, pancreas area, all that just generally in that area. I’m seeing a slow worsening at this point in time.” (Week 13)	No patients	“after I received my infusion I think it was a day or so after, I’m a little fatigued […] not very bad, not that I can’t’ do everything but I’d like to kind of back off that day and take it easy for that day” (Week 25)	“I’m seeing a slow worsening at this point in time. A little bit, like I say, a little more abdominal pain […] which I would grade now on a level of one to ten, probably in the 3 range, 4 range, when I have it, it’s not a continuous pain, it’s when I’m in certain positions like laying down in bed on my back, it will bother me.” (Week 13)

*N/A* not applicable as data is missing for the patient with progressive disease

^a^Clinical status, as defined based on the overall response by Independent Endpoint Review Committee per Response Evaluation Criteria In Solid Tumors version 1.1

^b^As categorized during qualitative analysis [35]

^C^patients answered to the question: “Has your cancer changed at all, since you started the study and received the study treatment?” during the follow-up interview

#### Perceived change in cancer since starting study treatment (Table 3)

At Week 13, seven out of eight patients whose tumour responded to avelumab treatment reported during their interview that they perceived a subjective improvement in cancer since starting study treatment. One patient had an improved tumour response but did not perceive any change in cancer. At Week 25, all the patients whose tumours responded to treatment (*n* = 8) perceived an improvement in their cancer since starting study treatment. The one patient with MCC progressing on treatment at both Week 13 and Week 25, reported their cancer had worsened between baseline and Week 13, then did not change between Week 13 and Week 25.

#### Physical functioning (Table 4)

At Week 13, among the eight patients with tumours responding to treatment, two perceived an improvement in their ability to perform activities compared to baseline, and two still noticed having to limit themselves. All four patients with improved physical functioning, and whose tumours were still responding to treatment at Week 25, described further improvements at Week 25, including having more endurance, and being able to return to exercising. Among those patients, all reported no deterioration in at least one of the FACT-M functioning-related scores, except for one patient. The two patients who described limitations at Week 13 no longer perceived limitations at Week 25. Three other patients out of the eight patients whose tumour responded to treatment did not notice a change within themselves - reporting no perceived physical impact either prior to or since starting the study treatment to Week 25. The last of the eight patients responding to treatment reported noticing a worsening ability to do activities at Week 13 compared to baseline, and an improvement at Week 25. This patient also reported improvement in both physical and functional scores at Week 25. The patient whose tumour did not respond to treatment reported no impact of MCC on physical functioning prior to receiving the study treatment, and did not notice a change since starting the study treatment. This is reflective of his/her report of deterioration in FACT-M physical well-being score at Week 25.

#### Fatigue (Table 5)

At Week 13, among the eight patients with tumours responding to treatment, two perceived an improvement in their fatigue levels compared with baseline, having more energy and feeling less tired than they recalled following chemotherapy; one of these two patients also reported having even more energy at Week 25. Data was missing for the other patient at Week 25. Three patients whose tumour responded to treatment did not perceive any change in their level of fatigue at Week 13: two were still experiencing the lack of endurance and the fatigue that they were experiencing prior to starting the study treatment; one was still experiencing no fatigue. At Week 25, two of these three patients were still feeling tired, and one reported having more energy in that he no longer had to motivate himself. The remaining three patients with tumour responding to treatment perceived a worsening of their energy and fatigue levels at Week 13, though two reported an improvement at Week 25; data was missing for the third patient. The one patient whose tumour did not respond to treatment mentioned being a little fatigued a day after receiving the infusion of the study treatment at Week 25. This patient also reported more fatigue in the FACT-M at Week 25. However, no trend could be drawn as no data was reported spontaneously, nor probed during the Week 13 interview.

#### Pain (Table 5)

Among the eight patients whose tumour responded to treatment, one reported having no remaining pain at Week 13 and Week 25. Those patients also reported no more pain in the FACT-M at Week 25. One patient reported that he/she could still feel some back pain, but said that the pain tended to decrease by Week 13, and the pain further decreased at Week 25. Five other patients who reported no pain at baseline did not perceive any changes since starting the study treatment, still feeling no pain at week 13; four of these patients again reported no pain at Week 25, and data was missing for one patient. The last responding patient experienced pain at Week 13 that was not experienced prior to starting treatment; the pain was not reported by the patient nor probed by the interviewer at Week 25. The patient whose tumour was progressing perceived a worsening in his/her pain at Week 13, which was still present at Week 25 as he/she reported in the FACT-M pain item.

## Discussion

MCC is a rare skin cancer for which there are no MCC-specific patient-reported outcome instruments to assess patient’s quality of life, thus limiting the possibility to provide evidence of the meaningfulness of treatment benefit from the patients’ perspective in a clinical trial. To provide some additional evidence, and to overcome the limitations related to the small sample size and challenges inherent to rare conditions, a mixed methods approach with a convergent design was used [37]. Patients were invited to participate in qualitative interviews as they consented to the JAVELIN Merkel 200 (NCT02155647) phase 2 clinical trial in metastatic MCC patients whose disease had progressed after last chemotherapy regimen [30], and qualitative findings were compared with the patients’ clinical status (i.e. CR, PR and PD). While a conceptual framework about the journey of patients with MCC has been developed based on the baseline interviews [30], for the purpose of this analysis, we selected specific concepts among those from the conceptual framework that would be of interest for clinicians, other stakeholders and health authorities [5, 34].

Only 19 patients accepted to be interviewed out of which only 9 made it to complete week 25 follow-up interviews. We acknowledge that the sample size is very limited, however in this context of a very rare and aggressive disease on which very little is known, we believe that our data, descriptive and exploratory, are worth communicating and hopefully will encourage others to do so in this field.

There was a high concordance found between clinical and patient-reported qualitative data. All patients who improved clinically also had a subjective perception of improvement in their disease since receiving avelumab. In addition, this subjective perception of improvement raised during qualitative interviews was translated into no deterioration of their disease in the patient-reported quantitative assessment (FACT-M items). The single patient with MCC progressing on treatment did not perceive improvement in his/her disease at Week 13 and not notice worsening at Week 25 of treatment. At Week 13, some patients had not perceived a benefit in their physical functioning, fatigue/energy and pain despite observed clinical improvements; however, at Week 25 most patients (*n* = 7) perceived benefits (qualitatively and quantitatively) consistent with their tumour response assessment. This delay between clinically detected response and patients’ perception in their everyday life suggests that extended follow-up is needed to capture full and meaningful treatment benefit when considering health-related quality of life endpoints.

The majority of interviewed patients had tumours that responded partially or completely to avelumab (*n* = 8) at Week 13 and remained so by Week 25. One patient had a tumour that did not respond to treatment. We acknowledge that a limitation of this analysis remains that the subjects interviewed post-baseline were more likely to be responders and hence likely to report positive experiences with the treatment. More patients were available and willing to participate in the follow-up interview if the patient was continuing in the study treatment, which resulted in patients with stable disease or response to treatment were more likely to be re-interviewed.

A majority of interviewed patients perceived an improved overall change in their cancer status (*n* = 7), and one patient reported no change. A single patient perceived worsening of their condition at Week 13, though by Week 25 all the patients reported an improvement in the overall change in their cancer since starting avelumab treatment. The majority of patients whose physical functioning, fatigue and pain were impaired when entering the study also noticed no deterioration (based on patient-reported quantitative assessment) or even an improvement (based on patient-reported qualitative assessment) in these domains at Week 25. Of note, most patients when discussing the change in their tumour linked their improvement to treatment referring to the clinical assessments (e.g. radiological scans) as they were provided with the results by their clinical team. These experiences may have impacted patients’ perceptions of their own improvement. In addition, the low number of patients whose tumours were not responding to treatment (i.e. PD, *n* = 1) limits definite correlation of findings regarding the impact of disease progression on PROs.

A larger number of patients with disease progression on treatment would have allowed a more accurate trend of the progression of the different concepts of interest to be obtained. However, this limitation is not directly related to the mixed method approach we describe herein.

Patient experience is a combination of their perception of the situation, of their environment and of clinical reality. We acknowledge that factors other than the cancer itself or the treatment could have had an influence on the patients’ feedback. Among these factors are the patients’ age, events that may have occurred between the interviews, and patients’ health status prior to receiving the first dose administration of the study treatment (e.g. lack of energy due to the previous chemotherapy treatment). Qualitative research has a high value for better understanding the burden of diseases on patients’ everyday lives, and for implementing the best management, treatment and care. This is particularly true for life-threatening and rare diseases where data are very sparse. Although integration and interpretation of such qualitative data can be challenging for the qualitative researcher, it can provide a way to understand and explain what is meaningful to patients. Besides patient experience based on qualitative interviews tend to show more positive results than patient-reported quantitative assessment. This can be explained by the positive impact of the interviewee to “speak in their own voice and express their own thoughts and feelings” [38]. One asset of mixed methods is that it can complement quantitative measures (here the FACT-M questionnaire and clinical objective response) of a condition with a patients’ subjective perception. Mixed methods consider not only the unique clinical experience of patients, but also their own characteristics including the age, familial environment, social environment, and comorbidities. In the future it is expected that mixed methods will mature, especially in terms of integrating qualitative and quantitative data in a systematic and complementary manner.

## Conclusion

This innovative mixed methods approach conducted within a clinical trial shows how qualitative data can complement quantitative clinical data. Hopefully, this study will promote the use of such an approach to overcome the hurdles associated with rare diseases when seeking to characterize patient definitions of meaningful treatment benefit.

## Abbreviations

COI: Concept of interest
EORTC QLQ-C30: EORTC-Quality of life -C30
FACT-G: Functional assessment of cancer therapy –general
FDA: Food and drug administration
IERC: Independent endpoint review committee
MCC: Merkel cell carcinoma
P/CR: Partial or complete responder
PD: Progressive disease
PD-L1: Programmed death ligand 1
PRO: Patient-reported outcome
RECIST: Response evaluation criteria in solid tumours version 1.1
SD: Stable disease
